# Whole-brain histogram analysis and top 20% gray and white matter ratio of amyloid positron emission tomography: A comparison with the centiloid scale

**DOI:** 10.1007/s12149-026-02218-9

**Published:** 2026-05-18

**Authors:** Ryo Yamakuni, Shinya Seino, Anna Yamaki, Atsushi Shima, Hironobu Ishikawa, Mitsunari Abe, Harumasa Takano, Nobukatsu Sawamoto, Hirofumi Sekino, Shiro Ishii, Kenji Fukushima, Naoyuki Ukon, Takashi Kasai, Jin Narumoto, Takashi Hanakawa, Hiroshi Ito, Parkinson’s, Alzheimer’s disease Dimensional Neuroimaging Initiative (PADNI), Yuji Takahashi, Noritaka Wakasugi, Atsushi Sekiguchi, Kyoji Okita, Takashi Sakamoto, Tadashi Tsukamoto, Yoshie Omachi, Yohei Mukai, Noriko Nishikawa, Toshiki Mizuno, Fukiko Morii-Kitani, Teruyuki Matsuoka, Fumitoshi Niwa, Masaki Kondo, Yoko Nakano, Shizuko Sugou, Kazuaki Kanai, Nozomu Matsuda, Toshiki Nakahara, Hirooki Yabe, Naoto Kobayashi, Hiroshi Hayashi, Sinobu Kawakakatsu, Tomohisa Okada, Takeshi Nii, Hiroyasu Ikeno, Nagara Tamaki, Tomoya Kotani, Jun Tazoe, Kentaro Akazawa, Kei Yamada, Yoshiki Endo, Shigeyasu Sugawara, Ayaka Nemoto, Minoru Oto, Yayoi Kurihara, Katuyuki Kikori, Hideaki Takasumi, Takashi Kanezawa, Emiya Koike, Kotaro Hattori, Yasuhiro Hashimoto, Shiho Ubukata, Toshiya Murai, Yohei Aoshima, Kazuaki Sajima, Hiroko Fukuoka, Shinichiro Mogi, Kasumi Hattori, Hiroshi Hoshino, Yuka Ueda, Yuya Hagane, Kazuko Kanno, Koichi Kato, Mayumi Inoue, Atsuko Inoue, Kenji Yoshinaga, Hiroki Togo, Kenji Hishikawa, Toma Matsushima, Koji Furukawa, Daisuke Kambe, Akira Nishida, Ikko Wada, Haruhi Sakamaki-Tsukita, Kenji Yoshimura, Yuta Terada, Yusuke Sakato, Kiyoaki Takeda, Masanori Sawamura, Etsuro Nakanishi, Yosuke Taruno, Hodaka Yamakado, Ryosuke Takahashi, Yasutaka Fushimi, Yuji Nakamoto, Daisuke Goto, Wataru Toda, Yuhei Mori

**Affiliations:** 1 Department of Radiology and Nuclear Medicine, School of Medicine, Fukushima Medical University, 1 Hikariga-Oka, Fukushima, 960-1295 Japan; 2 Department of Radiology, Fukushima Medical University Hospital, Fukushima, Japan; 3 Human Brain Research Centre, Kyoto University Graduate School of Medicine, Kyoto, Japan; 4 Integrative Brain Imaging Centre, National Centre of Neurology and Psychiatry, Tokyo, Japan; 5 Department of Human Health Sciences, Kyoto University Graduate School of Medicine, Kyoto, Japan; 6 Advanced Clinical Research Centre, Fukushima Medical University, Fukushima, Japan; 7 Department of Neurology, Graduate School of Medical Science, Kyoto Prefectural University of Medicine, Kyoto, Japan; 8 Department of Psychiatry, Graduate School of Medical Science, Kyoto Prefectural University of Medicine, Kyoto, Japan; 9 Department of Integrated Neuroanatomy and Neuroimaging, Kyoto University Graduate School of Medicine, Kyoto, Japan

**Keywords:** Centiloid scale, Whole‑brain histogram analysis, Positron emission tomography, Alzheimer’s disease, Amyloid PET

## Abstract

**Objective:**

To compare whole-brain histogram analysis (WBHA) of amyloid-β (Aβ) positron emission tomography (PET) using several magnetic resonance imaging (MRI)-based brain extraction (brain extraction tool [BET]) methods, to evaluate a novel quantitative analysis method, namely the Top 20% Gray and White Matter Ratio (GW-ratio), to determine appropriate thresholds, and to evaluate diagnostic performance in comparison with the Centiloid scale (CL).

**Methods:**

We analyzed structural MRI, ^18^F-flutemetamol amyloid PET images, and dementia severity scores (Global Clinical Dementia Rating [G-CDR] and Mini-Mental State Examination [MMSE]) of 262 participants. For WBHA, BET was performed using structural MRI and three different BET software programs, namely High-Definition-BET (HD-BET), FMRIB Software Library (FSL), and Statistical Parametric Mapping (SPM). Skewness and mode-to-mean ratio (MMR) were measured using brain-extracted PET. The Top 20% Map was generated from SPM BET images, separated into gray and white matter voxels, and their ratio was calculated as the GW-ratio. The CL value was computed using structural MRI. Aβ-negativity or positivity was visually determined.

**Results:**

Skewness from three BET methods was strongly negatively correlated with CL. Skewness (HD-BET) showed the strongest correlation (R = -0.9043). Moreover, the GW-ratio strongly correlated with CL (R = 0.8332), whereas MMR, particularly MMR (FSL) (R = 0.2112), showed poor correlation. All indicators significantly distinguished Aβ-negative from Aβ-positive visuals. CL had the highest area under the curve (AUC, 0.9959), followed by skewness (HD-BET) (0.9927), Top 20% GW-ratio (0.9872), and skewness (SPM) (0.9779), with no statistical difference between CL and skewness (HD-BET) (*P* = 0.5763). Based on mean and SD values from 118 cognitively unimpaired (G-CDR = 0, MMSE ≥ 28) and visually Aβ-negative participants, 95% cut-off limits for Aβ-negative individuals without dementia symptoms were: CL ≤ 12.9, skewness (HD-BET) ≥ 0.1769, MMR (HD-BET) ≤ 0.9372, skewness (FSL) ≥ 0.1819, MMR (FSL) ≤ 1.9132, skewness (SPM) ≥ -0.0382, MMR (SPM) ≤ 1.1274, and GW-ratio ≤ 0.1079.

**Conclusion:**

WBHA using MRI-based BET and GW-ratio showed strong correlations with CL and demonstrated a diagnostic performance comparable to that of CL.

**Supplementary Information:**

The online version contains supplementary material available at 10.1007/s12149-026-02218-9.

## Introduction

Amyloid positron emission tomography (PET) imaging plays a pivotal role in the clinical management and diagnosis of Alzheimer’s disease (AD). In routine practice, physicians determine the presence of amyloid-beta (Aβ) deposition based on tracer-specific visual interpretation criteria. Although expert physicians’ visual interpretation is reliable, quantitative scaling methods, such as the standardized uptake value ratio (SUVR) [1], Centiloid scale (CL) [2, 3], and Aβ load (Aβ_L_) [4, 5], can further enhance diagnostic accuracy. A previous study demonstrated that inter-reader agreement and diagnostic confidence significantly improved with the use of the CL [6].

To calculate SUVR, CL, and Aβ_L_, individual amyloid PET images must undergo a normalization process to be converted into the Montreal Neurological Institute (MNI) coordinate space. Although several normalization methods are available, including those based on structural magnetic resonance imaging (MRI) [7], thin-slice computed tomography (CT) [8], or PET imaging alone [9, 10], normalization failures can still occur, albeit uncommonly. Furthermore, SUVR and CL measure specific regions of interest, making it impossible to evaluate amyloid deposits outside these predefined volumes of interest (VOIs).

Okuyama et al. recently introduced a whole-brain histogram analysis (WBHA) and Top 20% Map method for amyloid PET [11]. Their approach involved removing the skull and skin from amyloid PET images using simultaneously acquired CT images. Subsequently, the skewness and mode-to-mean ratio (MMR) were calculated from the brain-extracted PET images. They also measured SUVR and demonstrated that skewness and SUVR exhibited a moderate correlation, whereas MMR and SUVR showed a strong correlation. Moreover, they developed the Top 20% Map display method, which highlights areas with high tracer accumulation occupying 20% of the total brain parenchymal volume to support radiographic interpretation. This method is advantageous because it eliminates the need for a normalization process, allows measurements in an individual’s native space, and does not require defining reference and target brain regions.

Although Okuyama’s method is reliable, it necessitates CT for brain extraction (brain extraction tool [BET]). However, since CT is not included in the Global Alzheimer’s Association Interactive Network (GAAIN) public dataset (https://www.gaain.org/centiloid-project), performing histogram analysis value calibration between tracers using this method is difficult. Moreover, previous studies have not determined threshold values for WBHA by correlating them with physicians’ visual assessments or indicators of dementia severity. Therefore, establishing MRI-based methods and determining thresholds are essential for the future development of histogram analysis.

Recently, several MRI-based BET software packages have become available with greatly improved performance. We also developed a novel quantitative method, the Top 20% Gray and White Matter Ratio (GW-ratio), extending the Top 20% Map approach. This study, therefore, aimed to establish an MRI-based WBHA method (including the Top 20% GW-ratio), compare histogram analysis values across BET methods, determine thresholds from a large database, and evaluate diagnostic performance against CL.

## Materials and methods

### Study design

This multicenter, cross-sectional, retrospective study was approved by the Institutional Ethics Committee of Fukushima Medical University (file number: REC2025—041). Given the anonymized nature of the data and retrospective study design, the requirement for written informed consent was waived. An opt-out approach was employed by publishing a summary of the study on the institutional websites. All procedures involving human participants were conducted in accordance with the ethical standards of the institutional and/or national research committees and according to the principles of the 1964 Helsinki Declaration and its later amendments or comparable ethical guidelines.

### Participants

All amyloid PET and structural MRI data from the Parkinson’s and Alzheimer’s Disease Dimensional Neuroimaging Initiative (PADNI; https://padni.org) were retrospectively and systematically reviewed. PADNI is a cohort study involving participants with AD, mild cognitive impairment (MCI), dementia with Lewy bodies (DLB), Parkinson’s disease (PD), and normal controls (NC) [12]. Participants were recruited from four institutions.

**Fig. 1:**
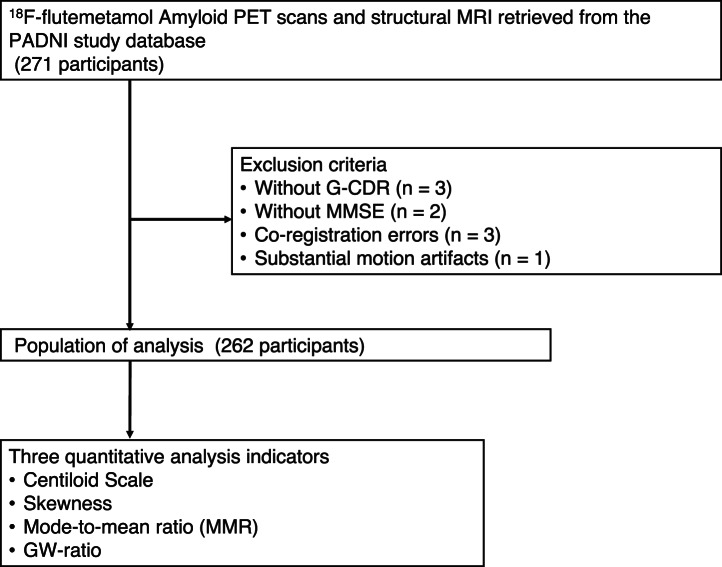
Flowchart of participants’ selection

In the PADNI study, amyloid PET imaging was performed using the following tracers: ^11^C-Pittsburgh compound B, ^18^F-florbetapir, and ^18^F-flutemetamol (FMM). Since each tracer has a distinct brain distribution pattern, histogram analysis can be affected by these differences in the distribution pattern [13]. Therefore, only participants who underwent ^18^F-FMM PET were included, because ^18^F-FMM was the most used tracer in the PADNI study. The earliest scan was selected when multiple PET or MRI scans were available for each participant. In addition, participants with substantial motion artefacts were excluded, as such artefacts can affect both the histogram analysis and CL measurements. The participant selection flowchart is provided in Fig. 1.

### Dementia severity

Dementia severity was assessed using scores from the Global Clinical Dementia Rating (G-CDR) [14] and Mini-Mental State Examination (MMSE) [15], both obtained from the PADNI database. The G-CDR is a 5-point scale that quantifies dementia severity as follows: 0 = normal, 0.5 = very mild, 1 = mild, 2 = moderate, and 3 = severe [14]. Consistent with previous studies, an MMSE cut-off score of 28 demonstrated optimal sensitivity and specificity for identifying MCI [16, 17]. Accordingly, participants in our study with a G-CDR score of 0 and an MMSE score of ≥ 28 were classified as having normal cognitive function. Participants lacking dementia severity data were excluded from the analysis.

### PET and structural MRI acquisition

In the PADNI study, a 20-min list-mode PET scan was performed 90 min after the injection of ^18^F-FMM. In addition, thin-slice sagittal T1-weighted imaging was performed to enable MRI-based spatial normalization and support BET for PET processing. The representative PET and MRI acquisition parameters are summarized in Table 1. In this study, the original PET count images were used as the PET images.

**Table 1:** Representative image collection and reconstruction method of amyloid PET and structural MRI

	Institution A	Institution B	Institution C	Institution D
*Amyloid PET*				
Scanner	Siemens Biograph mMR	GE Discovery IQ	Siemens Biograph Horizon 4R	Siemens Biograph 16 TruePoint
3D-PET acquisition	list mode	list mode	list mode	list mode
Acquisition time	20 min	20 min	20 min	20 min
Matrix size	172*172	128*128	256*256	336*336
Pixel size	4.17253 mm	1.953 mm	1.41015 mm	2.07 mm
Reconstruction method	3D OSEM	3D OSEM	3D OSEM	Iterative 3D
Iteration	3	5	8	4
Subset	21	12	10	21
Smoothing Filter	Gaussian 5 mm	Gaussian 4 mm	Gaussian 4 mm	All-pass
Attenuation correction	DIXON MRAC	CTAC	CTAC	CTAC
Scatter correction	model-based method	model-based method	relative	SSS
Randoms correction	DLYD	Singles Estimation	DLYD	DLYD
Slice thickness	2.03125 mm	3.26 mm	2 mm	2 mm
Field of view	58.8 cm	25.0 cm	36.1 cm	34.2 cm
*Structural MRI*				
Scanner	Siemens Biograph mMR	Siemens MAGNETOM Skyra	Siemens MAGNETOM Skyra	Siemens MAGNETOM Skyra fit
Sequence	3D-MPRAGE	3D-MPRAGE	3D-MPRAGE	3D-MPRAGE
Repetition time	1800 ms	2500 ms	2500 ms	2500 ms
Echo time	1.98 ms	2.18 ms	2.18 ms	2.18 ms
Inversion time	800 ms	1000 ms	1000 ms	1000 ms
Flip angle	9°	8°	8°	8°
Field of view	250 mm	256 × 240 × 179.2 mm	256 mm	240 × 256 × 180 mm
Acquisition matrix size	256 × 256	320 × 300 × 224	300 × 320	320 × 300 × 224
Slice thickness	1.0 mm	0.8 mm	0.8 mm	0.8 mm
Bandwidth	250 Hz/pixel	220 Hz/pixel	220 Hz/pixel	220 Hz/pixel

SSS, single scatter simulation; DLYD, delayed event subtraction; 3D-MPRAGE, three-dimensional magnetization-prepared rapid acquisition gradient-echo; 3D-SPACE, three-dimensional sampling perfusion with application-optimized contrasts by using different flip angle evolutions

### Visual Aβ-negative or Aβ-positive analysis

Visual analysis results were derived from those of a previous study that comprised two step visual analysis and determined the CL cut-off value using the PADNI amyloid PET dataset [18].

First, five nuclear medicine physicians assessed the images only visually (visual-only Aβ diagnosis). For each participant, two physicians were randomly assigned and blinded to the patient identification information and quantitative analysis results. They independently categorized each participant as Aβ-negative or positive based on tracer-specific criteria. In cases of disagreement, a third physician was randomly selected from among the remaining three, and the final classification was made by consensus.

Second, based on previously established thresholds [19], cases with a CL value < 10 classified as visually Aβ-positive (n = 1) and those with a CL value > 30 classified as Aβ-negative (n = 17), were extracted. For these discordant cases, an additional visual assessment was performed by two nuclear medicine physicians certified by the Japanese Society of Nuclear Medicine (JSNM), who had not participated in the initial visual-only Aβ diagnosis. These physicians, who had completed a JSNM-approved amyloid PET training course and possessed 23 and 10 years of experience, respectively, were provided with the CL values and independently re-evaluated each case as either Aβ-negative or Aβ-positive. Whenever their assessments differed, discrepancies were resolved via discussion. Using this approach, each case was ultimately classified as either Aβ-negative or Aβ-positive (final Aβ diagnosis).

### CL analysis

CL values were calculated using the Aβ PET analysis software Amyquant (version 1.00.0013; H. Matsuda, Tokyo, Japan), which utilizes the Statistical Parametric Mapping 12 (SPM12) processing pipeline [20]. In Amyquant, CL values were derived through the following steps. First, co-registration of the PET images to the structural MRI space was performed. Second, the structural MRI was normalized to the MNI coordinate space, and deformation fields were generated. Third, the co-registered PET images were transformed into the MNI space using the deformation fields obtained in the second step. Fourth, spatial smoothing was performed. Fifth, the global SUVR was calculated using the GAAIN cerebral cortical and striatal VOIs, with the entire cerebellar VOIs used as the reference region. Following the global SUVR calculation, CL values were computed using the following equation for ^18^F-FMM in the Amyquant software:

$$CL=-121.16+121.42\times {global SUVR}_{IND}$$

### Data pre-processing and brain extraction

Initially, the PET images were spatially aligned to the structural MRI space through co-registration and reslicing using SPM12 (https://www.fil.ion.ucl.ac.uk/spm/software/spm12/) [21]. Subsequently, spatial smoothing was applied to the co-registered images utilizing a Gaussian kernel with a 5 mm full width at half maximum (FWHM).

**Fig. 2:**
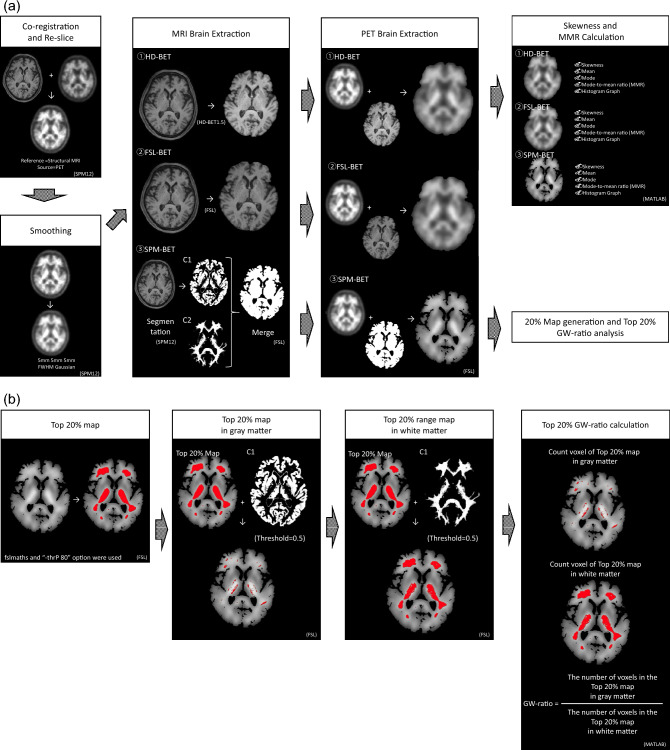
**(a)** Processing pipeline for quantitative whole-brain histogram and Top 20% Map analysis. The quantitative analysis pipeline consisted of the following sequential steps: 1) Image alignment and pre-processing: PET images were co-registered and resliced to the structural MRI space using Statistical Parametric Mapping (SPM) 12. Spatial smoothing was subsequently performed with a 5 mm Full FWHM Gaussian kernel. 2) Brain extraction (BET): Brain extraction was conducted on the structural MRI using three distinct BET methods. 3) PET masking: Brain extraction on the PET images was performed by masking the smoothed, resliced, and co-registered PET data with the brain-extracted structural MRI utilizing FSL software. 4) Histogram parameter calculation: Skewness and the Mode-to-Mean Ratio (MMR) were measured from the extracted PET data using MATLAB.** (b)** Top 20% Map generation and Top 20% GW-ratio analysis. 1) The Top 20% Map was generated using FSL. This map was then segmented into gray matter (GM) and white matter (WM) components based on tissue probability maps derived from the SPM BET results. 2) Top 20% GW-ratio calculation: Voxels in the separated Top 20% GM and WM maps were counted using MATLAB, and the Top 20% Gray and White Matter Ratio (GW-ratio) was subsequently calculated. Abbreviations**: **PET, Positron emission tomography; MRI, Magnetic resonance imaging; FWHM, Full width at half maximum; BET, brain extraction

Brain-extracted PET data were generated by performing BET on the structural MRI using three methods: High-Definition (HD)-BET, FMRIB Software Library (FSL), and SPM12. Further details regarding these specific BET software implementations are provided in the subsequent section. The smoothed, resliced, and co-registered PET data were then masked by the resultant brain-extracted MRI template to isolate the brain parenchyma. This masking operation was conducted using FSL (https://fsl.fmrib.ox.ac.uk) [22]. The comprehensive pipeline for PET brain extraction is illustrated in Fig. 2.

### HD-BET

Brain extraction was performed with the deep learning-based HD-BET software (version 1.5, available at https://github.com/MIC-DKFZ/HD-BET). This algorithm is generally reported to achieve superior accuracy compared to conventional BET methods, including FSL-BET, 3D-SkullStrip, BSE, ROBEX, BEaST, and MONSTR [23]. Although the software supports both Graphics Processing Unit and Central Processing Unit (CPU) modes, the CPU mode was selected for this study to ensure broad compatibility across different computing environments.

### FSL-BET

In the FSL-BET method, BET was performed using FSL software (version 6.0.7.17, https://fsl.fmrib.ox.ac.uk). The FSL-BET algorithm was executed with default settings, and no specific options for precision enhancement were applied.

### SPM-BET

For brain extraction with SPM, structural MRI was first processed using tissue segmentation in SPM12 (version 7771, https://www.fil.ion.ucl.ac.uk/spm/software/spm12/) [21]. This generated six tissue probability maps (c1–c6), corresponding to gray matter (GM, c1), white matter (WM, c2), cerebrospinal fluid (CSF, c3), bone (c4), soft tissue (c5), and background (c6). The final brain parenchyma mask was constructed by combining the GM (c1) and WM (c2) probability maps via FSL.

### Calculation of histogram parameters

Brain-extracted PET images were processed with a custom MATLAB 2025a (MathWorks Inc., Natick, MA, USA) script to calculate WBHA parameters and generate histograms. The script followed these steps: **(A)** Data Conversion and Extraction: Voxel values were extracted and ordered sequentially. **(B)** Normalization: Voxel values were normalized to a range of 0–1000. **(C)** MMR Preparation: Non-zero normalized voxel values were isolated and rounded to the nearest integer for mode calculation. **(D)** MMR Determination: Mode and mean were calculated from the prepared non-zero data, and MMR was subsequently computed.

$$MMR=\frac{Mode\ value}{Mean\ value}$$

**Fig. 3:**
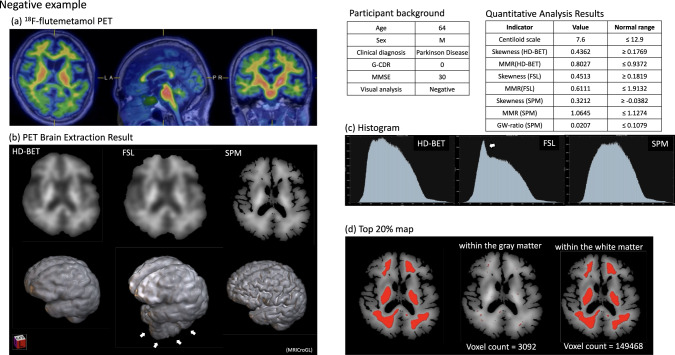
Representative images illustrating a 64-year-old male visually Aβ-negative case. **(a)** ^18^F‐flutemetamol PET/MRI, **(b)** PET BET result from three different BET methods, **(c)** Histogram from three BET methods, and **(d)** Top 20% map and voxel count result within gray matter and white matter. The PET BET results demonstrated that the FSL method failed to completely remove non-parenchymal tissue. The histogram generated using the FSL method also exhibits a low-uptake spectrum corresponding to non-brain parenchymal regions. Abbreviation**:** CL, Centiloid; BET, brain extraction

**Fig. 4:**
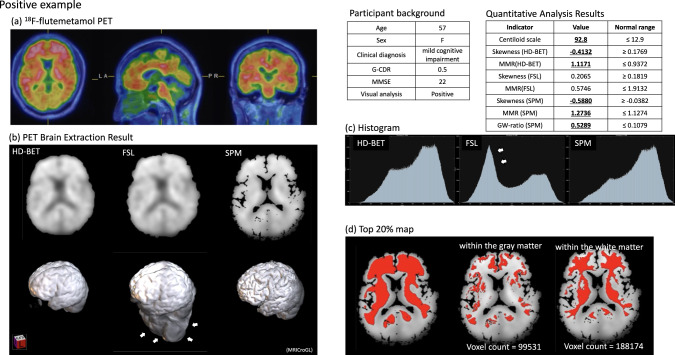
Representative images illustrating a 57-year-old female visually Aβ-positive case. **(a)** ^18^F‐flutemetamol PET/MRI, **(b)** PET BET result from three BET methods, **(c)** Histogram from three BET methods, and **(d)** Top 20% map and voxel count results within gray matter and white matter. The PET BET results demonstrated that the FSL method failed to completely remove non-parenchymal tissue. The histogram generated using the FSL method also exhibits a low-uptake spectrum corresponding to non-brain parenchymal regions. Abbreviation**:** CL, Centiloid; BET, brain extraction

**(E)** Skewness Calculation: Skewness was calculated separately using the original extracted non-zero voxel values. **(F)** Data Output and Visualization: The normalized non-zero voxel values (from the MMR Preparation step) were exported to a text file and then re-read to generate the final histogram. Figure 2a illustrates the comprehensive processing pipeline for WBHA parameter calculation. Figures 3 and 4 provides examples of typical histogram morphologies and the corresponding trends observed in the calculated MMR and skewness values.

In summary, WBHA parameters (specifically skewness and MMR) were systematically calculated for PET images that had undergone BET using the three software methods: HD-BET, FSL, and SPM12.

### Top 20% GW-ratio

Top 20% maps were generated from a brain-extracted PET image using SPM-BET (Fig. 2a). These maps were subsequently segmented into GM and WM components using tissue probability maps derived from the SPM processing, with a threshold set at 0.5. The resulting voxels in the separated Top 20% GM and WM maps were counted using MATLAB. The Top 20% GW-ratio was then calculated using the following formula:

$$\begin{aligned}&Top 20\% GW - ratio\\ &\quad =\frac{The\ number\ of\ voxels\ in\ the\ Top\ 20\%\ Map\ located\ within\ the\ Gray\ matter}{The\ number\ of\ voxels\ in\ the\ Top\ 20\%\ Map\ located\ within\ the\ White\ matter}\end{aligned}$$

Figure 2b illustrates the comprehensive processing pipeline for calculating the Top 20% GW-ratio.

### Statistical analysis

The correlations between CL and skewness and WBHA parameters and the Top 20% GW-ratio were investigated using simple correlation analysis (R), simple linear regression analysis, and the coefficient of determination (R^2^).

To estimate the normal range in participants without dementia symptoms, the mean and standard deviation (SD) of CL, skewness, MMR, and Top 20% GW-ratio were calculated from cognitively normal individuals, defined as those with no cognitive impairment (G-CDR = 0 and MMSE ≥ 28) and visually assessed as Aβ-negative. Based on these results, reference ranges were determined using the following formulas: 95% reference ranges = mean ± 2SD.

To predict visual Aβ diagnosis outcomes based on CL, skewness, MMR, and Top 20% GW-ratio, a receiver operating characteristic (ROC) curve was generated using binary logistic regression for these three quantitative analysis indicators. Moreover, ROC curves were created to differentiate cognitively normal participants (G-CDR = 0 and MMSE ≥ 28) from other groups. The optimal thresholds were determined from the ROC curves using the Youden index [24]. The areas under the curve (AUCs) were compared using the χ^2^ test.

Statistical analyses were performed using commercial software programs, including Bell Curve for Excel 4.09 (Social Survey Research Information, Tokyo, Japan) and Excel 2024 (Microsoft Corporation, Redmond, Washington, USA).

## Results

### Patient characteristics

Based on the exclusion criteria, three participants without G-CDR data, two without MMSE scores, three with co-registration errors, and one with substantial motion artefacts were excluded. The patient selection flowchart is shown in Fig. 1.

After applying the inclusion and exclusion criteria, 262 participants from the PADNI were included in the analyses. The participants comprised 137 males and 125 females, with a mean age of 69.6 years (range: 50–90 years). Each participant was clinically diagnosed with one of the following conditions: AD (n = 29), MCI (n = 71), DLB (n = 14), PD (n = 71), or was classified as a NC (n = 77). Among the 262 participants, 131 did not have cognitive impairments (G-CDR = 0 and MMSE ≥ 28), whereas 131 had cognitive impairments. The participants’ data are summarized in Table 2.

**Table 2:** The participants’ demographic information and dementia severity

Clinical diagnosis	Demographic information					Dementia severity			
	Sex			Age		G-CDR		MMSE	
	all	male	female	Mean	SD	Mean	SD	Mean	SD
AD	29	9	20	71.8	9.2	0.6	0.5	24.2	5.9
MCI	71	38	33	73.3	7.4	0.5	0.1	26.1	3.0
DLB	14	9	5	76.3	5.3	0.7	0.4	24.6	4.1
PD	71	36	35	66.8	7.5	0.1	0.2	28.6	1.8
NC	77	45	32	66.5	8.3	0.0	0.0	29.2	1.2
All	262	137	125	69.5	8.5	0.3	0.3	27.4	3.4

AD, Alzheimer’s disease; MCI, mild cognitive impairment; DLB, dementia with Lewy bodies; PD, Parkinson’s disease; NC, normal controls; MMR, mode-to-mean ratio; SD, standard deviation; G-CDR, Global Clinical Dementia Rating; MMSE, Mini-Mental State Examination;

### Visual analysis

Based on the initial visual-only Aβ diagnosis, 52 and 210 participants were classified as Aβ-positive and Aβ-negative, respectively. Discordance between the CL and initial visual-only results was observed in 18 cases. Specifically, one case was visually diagnosed as Aβ-positive despite a negative CL value (< 10), while the remaining 17 cases were visually diagnosed as Aβ-negative despite a positive CL value (> 30). A subsequent final Aβ diagnosis was performed in these 18 discordant cases, all of which reclassified as Aβ-positive. Consequently, of the 262 participants, the final visual analysis yielded 69 Aβ-positive and 193 Aβ-negative cases.

Supplementary Table 1 presents the 18 cases with discordance between the initial visual assessment and CL results that required re-evaluation, summarizing the CL and WBHA results for these discordant cases.

Among the 69 Aβ-positive participants, 56 had cognitive impairments (G-CDR > 0 and/or MMSE < 28). Among the 193 Aβ-negative participants, 118 had no cognitive impairments (G-CDR = 0 and MMSE ≥ 28). Figures 3 and 4 present Aβ-positive and negative images as well as a table of quantitative analysis indicators.

### Correlation among CL, skewness, MMR, and Top 20% GW-ratio

In simple correlation analysis, CL, skewness, MMR, and Top 20% GW-ratio were strongly correlated, except for MMR from FSL (Table 3). As shown in previous studies, skewness shows low values in amyloid-positive patients and consequently exhibits a negative correlation with CL and MMR. The Top 20% GW-ratio showed a positive correlation with CL and MMR, and a negative correlation with skewness.

**Table 3:** Simple correlation analysis among Centiloid scale, Skewness, MMR, and Top 20% GW-ratio

Correlation Coefficients	Centiloid Scale	Skewness (HD-BET)	MMR (HD-BET)	Skewness (FSL)	MMR (FSL)	Skewness (SPM)	MMR (SPM)
Skewness (HD-BET)	−0.9043						
MMR (HD-BET)	0.7735	−0.8396					
Skewness (FSL)	−0.8247	0.9287	−0.7799				
MMR (FSL)	0.2112	−0.2751	0.2825	−0.2859			
Skewness (SPM)	−0.8766	0.9287	−0.7736	0.8786	−0.2460		
MMR (SPM)	0.7265	−0.8159	0.6907	−0.7837	0.1666	−0.8410	
Top 20% GW-ratio (SPM)	0.8332	−0.8071	0.7129	−0.7858	0.2086	−0.8061	0.6383

95% Interval Estimation	**Centiloid Scale**	**Skewness (HD-BET)**	**MMR (HD-BET)**	**Skewness (FSL)**	**MMR (FSL)**	**Skewness (SPM)**	**MMR (SPM)**	**Top 20% GW-ratio (SPM)**
Centiloid Scale	-	−0.8795	0.8180	−0.7817	0.3241	−0.8452	0.7791	0.8668
Skewness (HD-BET)	−0.9242	-	−0.7998	0.9437	−0.1592	0.9437	−0.7709	−0.7603
MMR (HD-BET)	0.7197	−0.8721	-	−0.7275	0.3904	−0.7199	0.7491	0.7678
Skewness (FSL)	−0.8600	0.9100	−0.8233	-	−0.1706	0.9035	−0.7321	−0.7346
MMR (FSL)	0.0924	−0.3835	0.1671	−0.3934	-	−0.1286	0.2821	0.3216
Skewness (SPM)	−0.9019	0.9100	−0.8181	0.8476	−0.3565	-	−0.8015	−0.7591
MMR (SPM)	0.6637	−0.8527	0.6215	−0.8264	0.0464	−0.8732	-	0.7050
Top 20% GW-ratio (SPM)	0.7919	−0.8456	0.6477	−0.8282	0.0897	−0.8448	0.5605	-

(Upper Triangular: Upper Bound / Lower Triangular: Lower Bound)

**Fig. 5:**
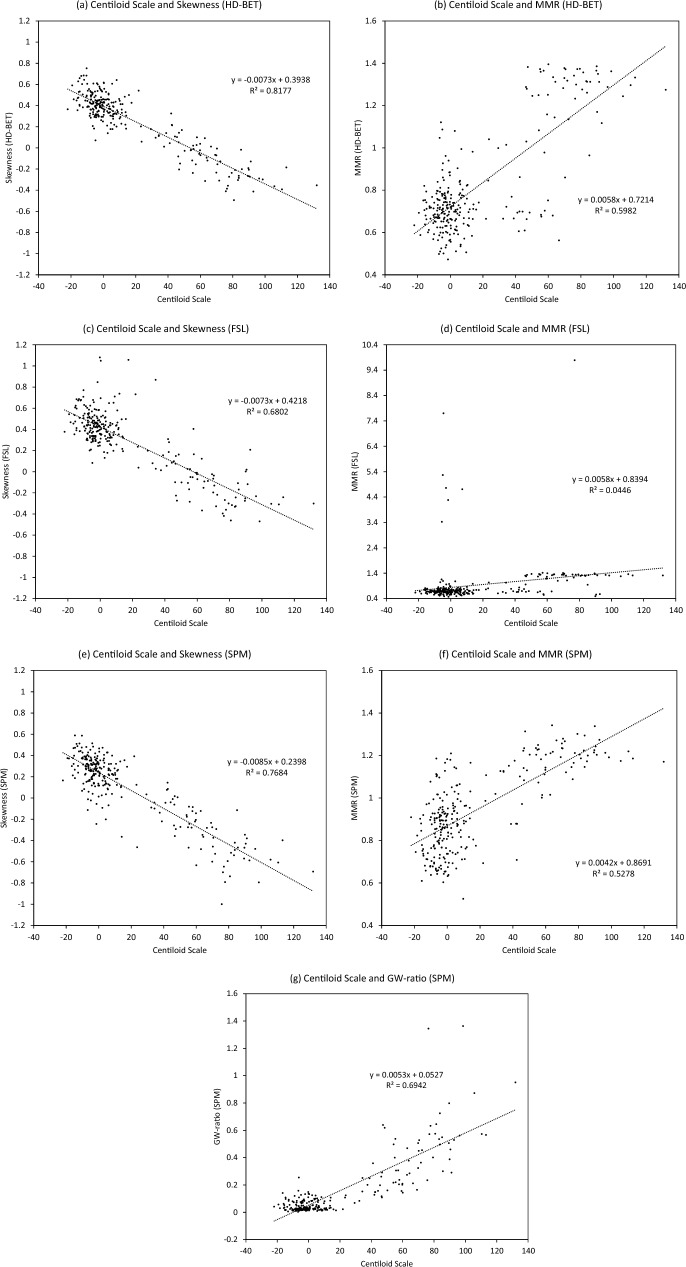
Correlation between CL and: **(a)** skewness (HD-BET), **(b)** MMR (HD-BET), **(c)** skewness (FSL), **(d)** MMR (FSL), **(e)** skewness (SPM), **(f)** MMR (SPM), and **(g)** Top 20% GW-ratio. Negative correlations were observed between CL and skewness. Positive correlations were observed between CL and MMR, and between CL and the Top 20% GW-ratio. Abbreviations**:** MMR, mode-to-mean ratio; CL, Centiloid; GW-ratio, Gray and White matter ratio

In simple regression analysis, CL, skewness, MMR, and Top 20% GW-ratio were strongly correlated, except MMR from FSL (Fig. 5). Among these quantitative analyses, skewness from HD-BET (R^2^ = 0.8177) showed the strongest correlation with CL, followed by skewness from SPM (R^2^ = 0.7684), and Top 20% GW-ratio (R^2^ = 0.6942).

### Normal range in participants without dementia symptoms and visually Aβ-negative

Among the 262 participants, 118 were cognitively unimpaired (G-CDR = 0, MMSE ≥ 28) and visually Aβ-negative. For this subgroup, the mean, SD, and 95% reference ranges for CL, skewness, MMR, and Top 20% GW-ratio are presented in Table 4.

**Table 4:** Normal Range in Participants without Dementia Symptoms and Visually Aβ-negative

Method	Mean	Standard deviation (SD)	reference ranges (mean ± 2SD)	95% cut-off limits (Normal)
Centiloid Scale	-1.5	7.2	(-15.9 to 12.9)	Centiloid Scale ≤ 12.9
Skewness (HD-BET)	0.4020	0.1125	(0.1769 to 0.6270)	Skewness (HD-BET) ≥ 0.1769
MMR (HD-BET)	0.7228	0.1072	(0.5084 to 0.9372)	MMR (HD-BET) ≤ 0.9372
Skewness (FSL)	0.4220	0.1200	(0.1819 to 0.6620)	Skewness (FSL) ≥ 0.1819
MMR (FSL)	0.7944	0.5594	(-0.3244 to 1.9132)	MMR (FSL) ≤ 1.9132
Skewness (SPM)	0.2580	0.1481	(-0.0382 to 0.5543)	Skewness (SPM) ≥ -0.0382
MMR (SPM)	0.8638	0.1318	(0.6003 to 1.1274)	MMR (SPM) ≤ 1.1274
Top 20% GW-ratio (SPM)	0.0417	0.0331	(-0.0244 to 0.1079)	Top 20% GW-ratio (SPM) ≤ 0.1079

Consistent with previous findings, higher CL and MMR values suggest more severe Aβ deposition, whereas lower skewness values reflect stronger Aβ deposition [11]. In amyloid PET imaging, tracer accumulation in WM is considered physiological, whereas considerable accumulation in the GM cortex indicates pathology. Consequently, an elevated Top 20% GW-ratio is considered pathological. The 95% cut-off limits for identifying Aβ-negative participants without dementia symptoms were: CL ≤ 12.9, skewness (HD-BET) ≥ 0.1769, MMR (HD-BET) ≤ 0.9372, skewness (FSL) ≥ 0.1819, MMR (FSL) ≤ 1.9132, skewness (SPM) ≥—0.0382, MMR (SPM) ≤ 1.1274, and Top 20% GW-ratio (SPM) ≤ 0.1079.

### ROC analysis

**Fig. 6:**
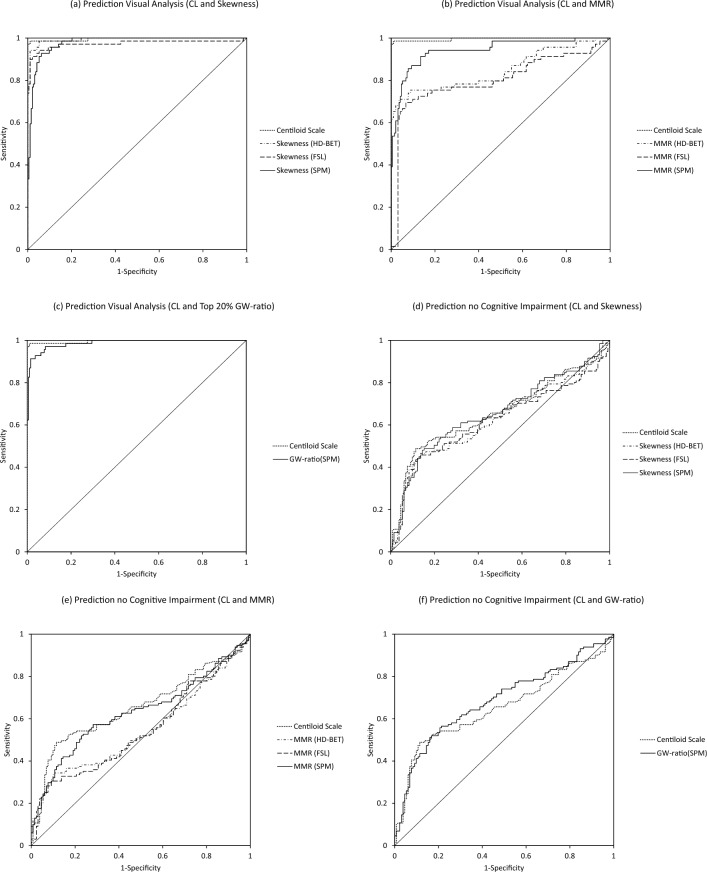
ROC curve analysis differentiating positive from negative visual Aβ diagnosis for: **(a)** Skewness, **(b)** MMR, **(c)** Top 20% GW-ratio compared with CL. ROC curve analysis differentiating cognitively normal participants (G-CDR = 0 and MMSE ≥ 28) from others for: **(d)** Skewness, **(e)** MMR, **(f)** Top 20% GW-ratio compared with CL. Abbreviations**:** ROC, Receiver operating characteristics; G-CDR, Global clinical dementia rating; MMSE, Mini-Mental State Examination; MMR, mode-to-mean ratio; GW-ratio, Gray and White matter ratio; CL, Centiloid

The AUCs for CL, skewness (HD-BET, FSL, SPM), MMR (HD-BET, FSL, SPM), and Top 20% GW-ratio were significant in distinguishing Aβ-positive from Aβ-negative visual Aβ diagnoses. CL showed the highest AUC (0.9959; 95% confidence interval [CI]: 0.9880–1.0037; *P* < 0.001). Among WBHA parameters, skewness (HD-BET) showed the highest AUC (0.9927; 95% CI: 0.9847–1.0007; *P* < 0.001), followed by Top 20% GW-ratio, skewness (SPM), and skewness (FSL). ROC results are presented in Fig. 6a–c and Table 5; AUC comparisons are in Supplemental Table 2.

**Table 5:** Logistic regression results and AUCs for assessing the utility of quantitative indicators in radiologist-diagnosed amyloid positivity/negativity

Method	AUC (95% CI)		Cutoff (Youden-Index)	Sensitivity (%)	Specificity (%)	Chi-square test
Centiloid Scale	0.9959	(0.9880 - 1.0037)	23.3	98.6	99.0	P < 0.001 **
Skewness (HD-BET)	0.9927	(0.9847 - 1.0007)	0.2173	98.6	94.8	P < 0.001 **
MMR (HD-BET)	0.8493	(0.7843 - 0.9143)	0.9643	71.0	96.4	P < 0.001 **
Skewness (FSL)	0.9730	(0.9423 - 1.0038)	0.1856	91.3	97.9	P < 0.001 **
MMR (FSL)	0.8020	(0.7285 - 0.8755)	0.9449	69.6	93.3	P < 0.001 **
Skewness (SPM)	0.9779	(0.9636 - 0.9922)	0.0172	92.8	93.8	P < 0.001 **
MMR (SPM)	0.9421	(0.9074 - 0.9768)	1.0014	91.3	86.5	P < 0.001 **
Top 20% GW-ratio (SPM)	0.9872	(0.9759 - 0.9768)	0.1433	91.3	98.4	P < 0.001 **

^*^ P < 0.05, **P < 0.01. AUC, area under the curve; MMR, mode-to-mean ratio

The AUCs for CL, skewness (HD-BET, FSL, SPM), MMR (SPM), and Top 20% GW-ratio were significant in predicting cognitively normal participants (G-CDR = 0 and MMSE ≥ 28) from other groups. The Top 20% GW-ratio showed the highest AUC (0.6863; 95% CI: 0.6211–0.7515; *P* < 0.001), followed by skewness (SPM), CL, and MMR (SPM). MMR (HD-BET) and MMR (FSL) were not significant. ROC results are presented in Fig. 6d–f and Table 6; AUC comparisons are presented in Supplemental Table 3.

**Table 6:** Logistic regression results and AUCs for differentiating cognitively normal participants (G-CDR = 0 and MMSE ≥ 28) from other groups using quantitative indicators

Method	AUC (95% CI)		Cutoff (Youden-Index)	Sensitivity (%)	Specificity (%)	Chi-square test
Centiloid Scale	0.6538	(0.5856 - 0.7219)	11.9	48.9	88.5	P < 0.001 **
Skewness (HD-BET)	0.6285	(0.5596 - 0.6975)	0.2187	46.6	86.3	P < 0.001 **
MMR (HD-BET)	0.5492	(0.4780 - 0.6975)	0.9268	34.4	89.3	0.1756
Skewness (FSL)	0.6181	(0.5479 - 0.6884)	0.1984	42.8	89.3	P < 0.001 **
MMR (FSL)	0.5446	(0.4738 - 0.6154)	1.0917	30.5	90.8	0.2169
Skewness (SPM)	0.6556	(0.5882 - 0.7230)	0.0589	48.1	85.5	P < 0.001 **
MMR (SPM)	0.6293	(0.5604 - 0.7230)	0.9376	57.3	71.8	P < 0.001 **
Top 20% GW-ratio (SPM)	0.6863	(0.6211 - 0.7515)	0.0833	56.5	78.6	P < 0.001 **

^*^ P < 0.05, **P < 0.01

AUC, area under the curve; MMR, mode-to-mean ratio

## Discussion

This study demonstrated strong correlations between CL and histogram analysis indicators using three different BET methods based on structural MRI. Skewness (HD-BET), skewness (SPM), and Top 20% GW-ratio exhibited considerable diagnostic performance, nearly equivalent to that of CL. However, MMR, especially MMR (FSL), showed poorer correlation with CL compared with the other indicators.

**Fig. 7:**
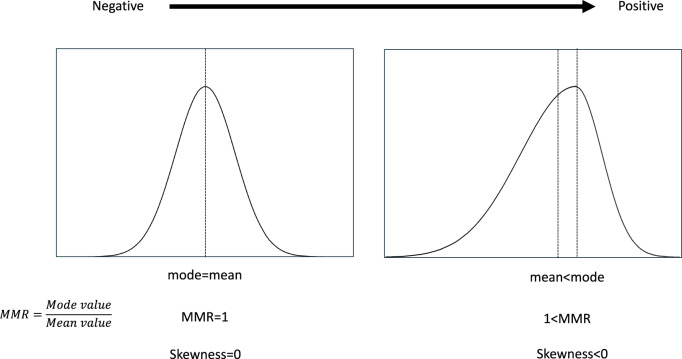
Changes in skewness and MMR associated with the histogram shift from amyloid-negative to amyloid-positive status

In the present study, skewness exhibited a negative correlation with CL, whereas MMR showed a positive correlation. These findings are consistent with the SUVR relationships reported in a previous WBHA study [11]. In healthy individuals without amyloid pathology, tracer uptake is limited to the WM, with no considerable accumulation in the cerebral cortex. This reflects the beta-sheet structure of myelin within the WM. In contrast, patients with amyloid pathology exhibit cortical uptake, which exceeds that of the WM, causing a rightward shift in the histogram. Consequently, as skewness decreases, the mode increases, and MMR becomes greater (Fig. 7). Therefore, in patients with AD, MMR is elevated while skewness is reduced. The negative and positive correlations between WBHA parameters and SUVR/CL observed in previous and present studies can be explained by these distributional changes.

In the current study, the Top 20% GW-ratio demonstrated a positive correlation with CL and MMR, while exhibiting a negative correlation with skewness. In healthy individuals, the Top 20% map displays a characteristic “kissing seahorses” appearance within the WM [11]. In amyloid-positive participants, cortical uptake exceeds physiological WM uptake. Consequently, the Top 20% map incorporates cortical regions, and the GW-ratio increases as the voxels within the Top 20% map become predominantly localized in the cortex, leading to higher values in amyloid-positive patients. This explains the observed correlations between the GW-ratio and both CL and WBHA parameters.

Among skewness values from the three BET methods, HD-BET showed the strongest correlation with CL, and the highest AUC. This may be attributed to the higher accuracy of HD-BET, a deep learning-based algorithm that outperformed five commonly used BET algorithms: FSL-BET, AFNI, Brainsuite, ROBEX, and BEaST [23]. Unlike other methods, HD-BET has a low probability of including non-brain tissues such as skull, fat, nasal, and orbital cavities, enabling accurate BET. Although SPM performed high-precision BET, its correlations with CL and AUC were lower than those of HD-BET, possibly due to partial volume effects. Differences in MRI and PET resolution may allow cortical uptake to spill over into regions identified as the brain sulcus on SPM. Strict removal of these sulcal regions may hinder accurate reflection of total cortical uptake. These findings suggest that a BET method with moderate precision, such as HD-BET, may be more effective for skewness, preserving relevant signal intensities.

Among MMR values from the three BET methods, HD-BET showed the strongest correlation with CL, whereas SPM demonstrated the highest AUC. This may relate to the potential removal of ventricles during SPM BET. As brain atrophy progresses, ventricular enlargement increases the volume of unremoved non-parenchymal tissue, influencing mean and mode values and potentially introducing bias favoring SPM. Further investigation is required to determine the optimal extent of CSF removal in WBHA.

Among the WBHA parameters, MMR consistently demonstrated inferior performance compared with skewness across all three BET methods. This discrepancy could potentially arise as MMR is more susceptible to image quality and noise than skewness. MMR is defined as the ratio of the mean to the mode. While the mean remains relatively stable despite noise from suboptimal image quality, the mode could be significantly altered by abnormalities in only a small fraction of pixels. Specifically, the estimation of the mode is inherently dependent on the histogram binning process, making it potentially more susceptible to local pixel value fluctuations compared with skewness, which integrates information across the entire distribution. For instance, even if 1,000 out of 100,000 pixels are abnormal, the mean remains substantially unaffected. However, if those 1,000 pixels converge on specific abnormal values, they might erroneously become the mode. In contrast, skewness characterizes the overall shape of the histogram distribution, rendering it more robust against localized noise. While both MMR and skewness could predict Aβ deposition, cases in which MMR is positive but skewness is negative should be carefully scrutinized for potential false positives. Further investigation would be required to identify the specific factors that influence MMR reliability.

WBHA offers several advantages over CL both in research and clinical settings. First, as spatial normalization is not required, processing could be performed within the native space of the participant, allowing for the direct use of the Top 20% Map for visual interpretation. Furthermore, normalization requirement elimination obviates the need for extensive computational resources, thereby reducing processing time. Although HD-BET was executed in the CPU mode in this study to ensure broader compatibility, switching to the GPU mode could further accelerate the process, enabling completion within seconds [23]. In addition, although not specifically addressed in this study, WBHA might facilitate quantification even in cases where spatial normalization is challenging, such as in patients with significant brain parenchyma loss due to stroke or tumors, which are frequently encountered in clinical practice, provided that appropriate BET is performed.

**Fig. 8:**
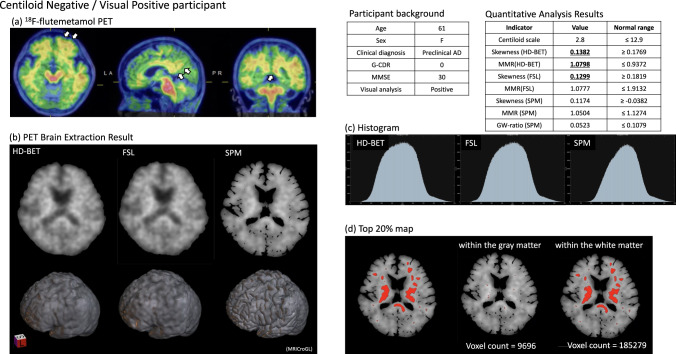
Case visually assessed as positive despite CL values within the normal range. Both skewness (HD-BET, FSL) and MMR (HD-BET) demonstrated values outside the reference range. **(a)** Amyloid accumulation was observed in the frontal lobe, posterior cingulate gyrus, and precuneus. **(b)** PET brain extraction yielded robust results. **(c)** Histogram exhibited a rightward shift relative to that in Fig. 3. **(d)** The Top 20% Map revealed scattered clusters, lacking the typical “kissing seahorses” appearance of normal cases

Second, WBHA does not require the definition of a reference brain region. CL cannot detect abnormal accumulation outside the GAAIN-defined regions of interest. In contrast, WBHA uses whole-brain data and can, in principle, detect pathological uptake beyond the GAAIN VOI. Consequently, it might detect cases in the so-called gray zone (CL 10–30) [25] or those where CL yields false negatives due to insufficient accumulation within the VOI. Indeed, in one case visually assessed as positive despite the CL values, both skewness (HD-BET, FSL) and MMR (HD-BET) were abnormal (Fig. 8). Although the CL generally reflected the final Aβ diagnosis more closely among the 18 discordant cases, partly because the second-step adjudication was triggered by the CL results themselves, the existence of this specific case suggests that WBHA could serve as a valuable complementary tool (Supplementary Table 1). Further research would be required to investigate the performance of WBHA in patients who are visually amyloid-positive but CL-negative.

Although WBHA can be performed in individual native spaces, another solution based on deep learning is available. For instance, Yamao et al. developed a deep learning method for directly predicting the CL from amyloid PET images in the native space [26]. Their study showed a strong correlation (R = 0.957) between the CL calculated using conventional methods and the CL predicted using the deep-learning method in ^18^F-FMM PET. However, the rationale behind deep-learning-based predictions is often difficult to interpret. Therefore, from a quantitative standpoint, the method presented in our study is considered superior to CL estimation using deep learning. Further studies are warranted to validate the quantitative indicators derived from analyses conducted in individual native spaces.

The strength of this study lies in its novel focus on the differences and correlations between CL and histogram analyses, including the determination of optimal thresholds. Furthermore, it demonstrates the feasibility of MRI-based WBHA, proposes the "Top 20% GW-ratio" as a novel extension of the Top 20% Map, and evaluates its diagnostic performance.

However, this study has some limitations. First, direct comparisons with BET using CT are lacking [27]. This is because the closed-source analysis software required for CT-based BET and histogram analysis in Okuyama’s method—the SYNAPSE VINCENT medical imaging system (FUJIFILM Medical, Tokyo, Japan)—was not available at our institution. In addition, the PADNI cohort included participants who underwent both PET/CT and PET/MRI; in several cases, CT images were not acquired simultaneously with PET scans. Second, while WBHA was useful for assisting in the determination of amyloid status in cases where CL and visual findings diverged or in gray-zone cases (CL = 10–30), this could not be definitively proven in the current study. Since definitive diagnosis of amyloid deposition is established by brain pathology, establishing a gold standard for living participants remains difficult, necessitating future reader experiments or studies using open data with pathological information. Third, this study was conducted using only 18F − FMM; since brain distribution varies by tracer, further research utilizing open-access databases such as GAAIN is required to determine tracer-specific normal values. Overall, further studies are warranted on histogram-based amyloid PET analysis, as it represents a valuable analytical method that can be performed within individual native spaces.

In conclusion, the WBHA of amyloid PET, particularly skewness from HD-BET or SPM, and the Top 20% GW-ratio, showed a strong correlation with CL and demonstrated a diagnostic performance comparable to that of CL. Conducting amyloid PET analysis within individualized native spaces may offer clinical utility, but further studies are warranted.

## Supplementary Information

Below is the link to the electronic supplementary material.

Supplementary file1 (ZIP 4 KB)

Supplementary file2 (PDF 123 KB)

Supplementary file3 (PDF 75 KB)

## Declarations

### Data Avilability

Data supporting the findings of this study are available from the corresponding author [R.Y.] upon reasonable request.
