# Supplementary material for: Whole-brain histogram analysis and top 20% gray and white matter ratio of amyloid positron emission tomography: A comparison with the centiloid scale
Source: Ann Nucl Med. 2026 May 18;40(8):983–98. doi: 10.1007/s12149-026-02218-9 (PMC13388449; doi:10.1007/s12149-026-02218-9)
Supplement: Supplementary file 2 — Supplementary file2 (PDF 123 KB) [file 12149_2026_2218_MOESM2_ESM.pdf]

Supplemental Table 1. Cases with discordance between visual only Aβ-positive and negative analysis and Centiloid Scale results requiring re-evaluation. This table summarizes Centiloid Scale values and whole-brain histogram analysis results for the discordant cases.

| No. | Age | Sex | MMSE | CDR (Global) | Centiloid Scale | Skewness (HD-BET) | MMR (HD-BET) | Skewness (FSL) | MMR (FSL) | Skewness (SPM) | MMR (SPM) | GW-ratio (SPM) | Visual-only Aβ diagnosis | Final visual Aβ diagnosis |
|---|---|---|---|---|---|---|---|---|---|---|---|---|---|---|
| | | | | | Normal Range | ≥ 0.1769 | ≤ 0.9372 | ≥ 0.1819 | ≤ 1.9132 | ≥ -0.0382 | ≤ 1.1274 | ≤ 0.1079 | | |
| 1 | 81 | male | 30 | 0 | **37.6** | **0.0111** | 0.7687 | **0.0129** | 0.7820 | **-0.1386** | **1.1516** | **0.2007** | NEGATIVE | POSITIVE |
| 2 | 66 | female | 29 | 0 | **42.6** | 0.2133 | 0.8616 | **0.2788** | 0.8347 | 0.0849 | 0.8782 | **0.1479** | NEGATIVE | POSITIVE |
| 3 | 90 | female | 17 | 1 | **57.2** | **-0.1565** | **1.3645** | **-0.0530** | 1.4141 | **-0.2787** | **1.1821** | **0.3067** | NEGATIVE | POSITIVE |
| 4 | 68 | female | 29 | 0.5 | **54.1** | **-0.0228** | **1.2491** | **0.0252** | 0.6473 | **-0.1607** | **1.2348** | **0.2165** | NEGATIVE | POSITIVE |
| 5 | 72 | male | 28 | 0.5 | **38.9** | **0.1396** | 0.6661 | **0.1547** | 0.6886 | **-0.2199** | 0.8777 | **0.2484** | NEGATIVE | POSITIVE |
| 6 | 82 | male | 26 | 0.5 | **42.4** | 0.2173 | 0.6990 | 0.1856 | 0.7438 | 0.1441 | 0.7084 | **0.1476** | NEGATIVE | POSITIVE |
| 7 | 79 | male | 30 | 0.5 | **45.6** | **0.1013** | 0.6092 | **0.0983** | 0.7731 | -0.0103 | 0.9717 | **0.1977** | NEGATIVE | POSITIVE |
| 8 | 66 | male | 26 | 0.5 | **42** | 0.3250 | 0.6057 | 0.3075 | 0.6634 | 0.0761 | 0.8791 | **0.1281** | NEGATIVE | POSITIVE |
| 9 | 76 | female | 24 | 0.5 | **32.4** | **0.0960** | 0.6652 | **0.0795** | 0.6615 | **-0.0901** | 1.1265 | 0.0833 | NEGATIVE | POSITIVE |
| 10 | 75 | female | 28 | 0.5 | **46.8** | **0.1422** | 0.6629 | **0.1481** | 0.6633 | 0.0172 | 1.0834 | **0.1086** | NEGATIVE | POSITIVE |
| 11 | 72 | male | 23 | 0.5 | **47.5** | **-0.1486** | 1.3821 | **-0.2743** | 1.3330 | **-0.2930** | **1.3132** | **0.6393** | NEGATIVE | POSITIVE |
| 12 | 77 | female | 27 | 0 | **41.1** | **0.0439** | 0.6936 | **0.0547** | 0.6553 | **-0.0454** | **1.1754** | **0.3584** | NEGATIVE | POSITIVE |
| 13 | 77 | male | 29 | 0.5 | **59.9** | **0.0385** | 0.7514 | **-0.0246** | 1.1870 | **-0.1439** | **1.1432** | **0.2086** | NEGATIVE | POSITIVE |
| 14 | 67 | female | 30 | 0 | **34.4** | **0.1193** | 0.7213 | **0.0273** | 1.0247 | **-0.0992** | 1.1231 | **0.2509** | NEGATIVE | POSITIVE |
| 15 | 74 | male | 28 | 0.5 | **66.6** | **0.0119** | 0.5624 | **-0.0882** | 1.1005 | **-0.2816** | **1.2709** | **0.2117** | NEGATIVE | POSITIVE |
| 16 | 81 | male | 26 | 0.5 | **57.7** | **0.0269** | **0.9779** | 0.4040 | 0.5722 | **-0.1237** | 1.0014 | **0.2047** | NEGATIVE | POSITIVE |
| 17 | 73 | male | 27 | 0.5 | **34.3** | **0.1119** | **1.0142** | 0.8680 | 0.6206 | **-0.0431** | 1.1269 | **0.1521** | NEGATIVE | POSITIVE |
| 18 | 61 | female | 30 | 0 | 2.8 | **0.1382** | **1.0798** | **0.1299** | 1.0777 | 0.1174 | 1.0504 | 0.0523 | POSITIVE | POSITIVE |

Bold and underlined text indicates values outside the normal range.

Supplemental Table 2. Comparison of Area Under the Curve (AUC) values to assess the utility of quantitative indicators for differentiating radiologist-diagnosed amyloid positivity from negativity.

| **Comparison of AUCs (Chi-square test)** | | **P-value** | |
|---|---|---|---|
| Centiloid Scale | Skewness (HD-BET) | 0.5763 | |
| Centiloid Scale | MMR (HD-BET) | P < 0.001 | ** |
| Centiloid Scale | Skewness (FSL) | 0.1595 | |
| Centiloid Scale | MMR (FSL) | P < 0.001 | ** |
| Centiloid Scale | Skewness (SPM) | 0.0141 | * |
| Centiloid Scale | MMR (SPM) | 0.0028 | ** |
| Centiloid Scale | Top 20% GW-ratio (SPM) | 0.0331 | * |
| Skewness (HD-BET) | MMR (HD-BET) | P < 0.001 | ** |
| Skewness (HD-BET) | Skewness (FSL) | 0.2028 | |
| Skewness (HD-BET) | MMR(FSL) | P < 0.001 | ** |
| Skewness (HD-BET) | Skewness (SPM) | 0.0242 | * |
| Skewness (HD-BET) | MMR (SPM) | 0.0014 | ** |
| Skewness (HD-BET) | Top 20% GW-ratio (SPM) | 0.4011 | |
| MMR (HD-BET) | Skewness (FSL) | P < 0.001 | ** |
| MMR (HD-BET) | MMR (FSL) | 0.2244 | |
| MMR (HD-BET) | Skewness (SPM) | P < 0.001 | ** |
| MMR (HD-BET) | MMR (SPM) | 0.0024 | ** |
| MMR (HD-BET) | Top 20% GW-ratio (SPM) | P < 0.001 | ** |
| Skewness (FSL) | MMR (FSL) | P < 0.001 | ** |
| Skewness (FSL) | Skewness (SPM) | 0.7652 | |
| Skewness (FSL) | MMR (SPM) | 0.1629 | |
| Skewness (FSL) | Top 20% GW-ratio (SPM) | 0.3919 | |
| MMR (FSL) | Skewness (SPM) | P < 0.001 | ** |
| MMR(FSL) | MMR (SPM) | P < 0.001 | ** |
| MMR (FSL) | Top 20% GW-ratio (SPM) | P < 0.001 | ** |
| Skewness (SPM) | MMR (SPM) | 0.0130 | * |
| Skewness (SPM) | Top 20% GW-ratio (SPM) | 0.2170 | |
| MMR (SPM) | Top 20% GW-ratio (SPM) | 0.0111 | * |

* P < 0.05, **P < 0.01

AUC, area under the curve; MMR, mode-to-mean ratio

Supplemental Table 3. Comparison of Area Under the Curve (AUC) values to assess the utility of quantitative indicators for differentiating cognitively normal participants (G-CDR = 0 and MMSE ≥ 28) from other groups using quantitative indicators.

| **Comparison of AUCs (Chi-square test)** | | **P-value** | |
|---|---|---|---|
| Centiloid Scale | Skewness (HD-BET) | 0.3338 | |
| Centiloid Scale | MMR (HD-BET) | 0.0052 | ** |
| Centiloid Scale | Skewness (FSL) | 0.2237 | |
| Centiloid Scale | MMR (FSL) | 0.0075 | ** |
| Centiloid Scale | Skewness (SPM) | 0.9448 | |
| Centiloid Scale | MMR (SPM) | 0.4386 | |
| Centiloid Scale | Top 20% GW-ratio (SPM) | 0.3076 | |
| Skewness (HD-BET) | MMR (HD-BET) | 0.0102 | * |
| Skewness (HD-BET) | Skewness (FSL) | 0.4434 | |
| Skewness (HD-BET) | MMR(FSL) | 0.0183 | * |
| Skewness (HD-BET) | Skewness (SPM) | 0.1460 | |
| Skewness (HD-BET) | MMR (SPM) | 0.9737 | |
| Skewness (HD-BET) | Top 20% GW-ratio (SPM) | 0.0290 | * |
| MMR (HD-BET) | Skewness (FSL) | 0.0362 | * |
| MMR (HD-BET) | MMR (FSL) | 0.8735 | |
| MMR (HD-BET) | Skewness (SPM) | 0.0032 | ** |
| MMR (HD-BET) | MMR (SPM) | 0.0183 | * |
| MMR (HD-BET) | Top 20% GW-ratio (SPM) | P < 0.001 | ** |
| Skewness (FSL) | MMR (FSL) | 0.0296 | * |
| Skewness (FSL) | Skewness (SPM) | 0.0711 | |
| Skewness (FSL) | MMR (SPM) | 0.6593 | |
| Skewness (FSL) | Top 20% GW-ratio (SPM) | 0.0051 | ** |
| MMR (FSL) | Skewness (SPM) | 0.0043 | ** |
| MMR(FSL) | MMR (SPM) | 0.0300 | * |
| MMR (FSL) | Top 20% GW-ratio (SPM) | P < 0.001 | ** |
| Skewness (SPM) | MMR (SPM) | 0.1826 | |
| Skewness (SPM) | Top 20% GW-ratio (SPM) | 0.2055 | |
| MMR (SPM) | Top 20% GW-ratio (SPM) | 0.0410 | * |

* P < 0.05, **P < 0.01

AUC, area under the curve; MMR, mode-to-mean ratio
