# Supplementary material for: Whole-brain histogram analysis and top 20% gray and white matter ratio of amyloid positron emission tomography: A comparison with the centiloid scale
Source: Ann Nucl Med. 2026 May 18;40(8):983–98. doi: 10.1007/s12149-026-02218-9 (PMC13388449; doi:10.1007/s12149-026-02218-9)
Supplement: Supplementary file 3 — Supplementary file3 (PDF 75 KB) [file 12149_2026_2218_MOESM3_ESM.pdf]

Supplementary information. Parkinson's and Alzheimer's disease Dimensional Neuroimaging Initiative (PADNI)

Executive Steering Committee:

Takashi Hanakawa, Principal Investigator

Clinical Core:

Takashi Hanakawa, Principal Investigator, Yuji Takahashi, Mitsunari Abe, Noritaka Wakasugi, Atsushi Sekiguchi, Harumasa Takano, Kyoji Okita, Takashi Sakamoto, Tadashi Tsukamoto, Yoshie Omachi, Yohei Mukai, National Center of Neurology and Psychiatry, Tokyo, Japan;

Noriko Nishikawa, Juntendo University School of Medicine, Tokyo, Japan;

Toshiki Mizuno, Fukiko Morii-Kitani, Takashi Kasai, Department of Neurology, Graduate School of Medical Science, Kyoto Prefectural University of Medicine, Kyoto, Japan;

Teruyuki Matsuoka, Jin Narumoto, Department of Psychiatry, Graduate School of Medical Science, Kyoto Prefectural University of Medicine, Kyoto, Japan;

Fumitoshi Niwa, Department of General Medicine and Medical Education, Graduate School of Medical Science, Kyoto Prefectural University of Medicine, Kyoto, Japan;

Masaki Kondo, Kyoto prefectural rehabilitation supporting center, Kyoto, Japan;

Yoko Nakano, Shizuko Sugou, Clinical Research Center, Kyoto Prefectural University of Medicine, Kyoto, Japan;

Atsushi Shima, Human Brain Research Center, Kyoto University Graduate School of Medicine, Kyoto, Japan;

Nobukatsu Sawamoto, Department of Human Health Sciences, Kyoto University Graduate School of Medicine, Kyoto, Japan;

Kazuaki Kanai, Nozomu Matsuda, Toshiki Nakahara, Department of Neurology , Fukushima Medical University School of Medicine, Fukushima, Japan;

Hirooki Yabe, Naoto Kobayashi, Department of Neuropsychiatry, Fukushima Medical University School of Medicine, Fukushima, Japan;

Hiroshi Hayashi, Department of Occupational Therapy, Fukushima Medical University School of Health Sciences, Fukushima, Japan;

Sinobu Kawakakatsu, Department of Neuropsychiatry, Fukushima Medical Universit Aizu Medical Center , Fukushima, Japan;

Imaging Core:

Tomohisa Okada, Department of Diagnostic Imaging and Nuclear Medicine, Kyoto University Graduate School of Medicine, Kyoto, Japan;

Harumasa Takano, Noritaka Wakasugi, Mitsunari Abe, National Center of Neurology and Psychiatry, Tokyo, Japan;

Takeshi Nii, Hiroyasu Ikeno, Department of Radiological Technology, University Hospital Kyoto Prefectural University of Medicine, Kyoto, Japan;

Nagara Tamaki, Tomoya Kotani, Jun Tazoe, Kentaro Akazawa, Kei Yamada, Department of Radiology, Graduate School of Medical Science, Kyoto Prefectural University of Medicine, Kyoto, Japan;

Hiroshi Ito, Shiro Ishii, Ryo Yamakuni, Yoshiki Endo, Anna Yamaki, Department of Radiology and Nuclear Medicine, Fukushima Medical University School of Health Sciences, Fukushima, Japan;

Shigeyasu Sugawara, Naoyuki Ukon, Ayaka Nemoto, Minoru Oto, Yayoi Kurihara, Advanced Clinical Research Center, Fukushima Global Medical Science Center, Fukushima Medical University, Fukushima, Japan;

Shinya Seino, Katuyuki Kikori, Hideaki Takasumi, Hironobu Ishikawa, Takashi Kanezawa, Emiya Koike, Department of Radiology, Fukushima Medical University Hospital, Fukushima, Japan

Statistics Core:

Takashi Hanakawa, Principal Investigator, Kenji Hatano, National Center of Neurology and Psychiatry, Tokyo, Japan;

Kazuaki Kanai, Department of Neurology , Fukushima Medical University School of Medicine, Fukushima, Japan;

Hirooki Yabe, Department of Neuropsychiatry, Fukushima Medical University School of Medicine, Fukushima, Japan;

Hiroshi Ito, Ryo Yamakuni, Department of Radiology and Nuclear Medicine, Fukushima Medical University School of Health Sciences, Fukushima, Japan;

BioRepository:

Kotaro Hattori, National Center of Neurology and Psychiatry, Tokyo, Japan;

Bioanalytics Core:

Kotaro Hattori, National Center of Neurology and Psychiatry, Tokyo, Japan;

Genetics Core:

Yuji Takahashi, National Center of Neurology and Psychiatry, Tokyo, Japan;

Yasuhiro Hashimoto, Department of Neurosurgery, and Department of Hygiene and Preventive Medicine, Fukushima Medical University School of Health Sciences, Fukushima, Japan;

Neuropsychological and Cognitive Assessments:

Shiho Ubukata, Toshiya Murai, Department of Psychiatry, Kyoto University Graduate School of Medicine, Kyoto, Japan;

Atsushi Shima, Human Brain Research Center, Kyoto University Graduate School of Medicine, Kyoto, Japan;

Noritaka Wakasugi, Yohei Aoshima, Kazuaki Sajima, Hiroko Fukuoka, Shinichiro Mogi, National Center of Neurology and Psychiatry, Tokyo, Japan;

Toshiki Nakahara, Kasumi Hattori, Department of Neurology , Fukushima Medical University School of Medicine, Fukushima, Japan;

Hiroshi Hoshino, Yuka Ueda, Yuya Hagane, Kazuko Kanno, Department of Neuropsychiatry, Fukushima Medical University School of Medicine, Fukushima, Japan;

Site Investigators and Coordinators:

Yuji Takahashi, Koichi Kato, Noritaka Wakasugi, Atsushi Sekiguchi, Yohei Aoshima, Kazuaki Sajima, Mayumi Inoue, Atsuko Inoue, Hiroko Fukuoka, Shinichiro Mogi, Kenji Yoshinaga, Hiroki Togo, Kenji Hishikawa, Toma Matsushima, National Center of Neurology and Psychiatry, Tokyo, Japan;

Koji Furukawa, Daisuke Kambe, Akira Nishida, Ikko Wada, Haruhi Sakamaki-Tsukita, Kenji Yoshimura, Yuta Terada, Yusuke Sakato, Kiyoaki Takeda, Masanori Sawamura, Etsuro Nakanishi, Yosuke Taruno, Hodaka Yamakado, Ryosuke Takahashi, Department of Neurology, Kyoto University Graduate School of Medicine, Kyoto, Japan;

Atsushi Shima, Human Brain Research Center, Kyoto University Graduate School of Medicine, Kyoto, Japan;

Yasutaka Fushimi, Tomohisa Okada, Yuji Nakamoto, Department of Diagnostic Imaging and Nuclear Medicine, Kyoto University Graduate School of Medicine, Kyoto, Japan;

Nobukatsu Sawamoto, Department of Human Health Sciences, Kyoto University Graduate School of Medicine, Kyoto, Japan;

Daisuke Goto, Wataru Toda, Yuhei Mori, Department of Neuropsychiatry, Fukushima Medical University School of Medicine, Fukushima, Japan;

Kasumi Hattori, Department of Neurology , Fukushima Medical University School of Medicine, Fukushima, Japan;

Conflict of interest of the PADNI group:

One of the group authors, R Takahashi received a research grant from Nihon Medi-Physics Co., Ltd., manufacturer of 123I-Ioflupane.

The remaining authors have nothing to report.
